# Physiological Adaptation to Water Salinity in Six Wild Halophytes Suitable for Mediterranean Agriculture

**DOI:** 10.3390/plants10020309

**Published:** 2021-02-05

**Authors:** Roberta Calone, Simone Bregaglio, Rabab Sanoubar, Enrico Noli, Carla Lambertini, Lorenzo Barbanti

**Affiliations:** 1 DISTAL—Dipartimento di Scienze e Tecnologie Agro-alimentari, University of Bologna, Viale Fanin 44, 40127 Bologna, Italy; rabab.sanoubar@poliambulatoriocavour.it (R.S.); enrico.noli@unibo.it (E.N.); carla.lambertini@unibo.it (C.L.); lorenzo.barbanti@unibo.it (L.B.); 2 CREA—Council for Agricultural Research and Economics, Research Centre for Agriculture and Environment, via di Corticella 133, 40128 Bologna, Italy; simoneugomaria.bregaglio@crea.gov.it

**Keywords:** halophytes, salt stress, salinity levels, relative water content, water potential

## Abstract

Owing to the high interspecific biodiversity, halophytes have been regarded as a tool for understanding salt tolerance mechanisms in plants in view of their adaptation to climate change. The present study addressed the physiological response to salinity of six halophyte species common in the Mediterranean area: *Artemisia absinthium, Artemisia vulgaris, Atriplex halimus, Chenopodium album, Salsola komarovii*, and *Sanguisorba minor*. A 161-day pot experiment was conducted, watering the plants with solutions at increasing NaCl concentration (control, 100, 200, 300 and 600 mM). Fresh weight (FW), leaf stomatal conductance (GS), relative water content (RWC) and water potential (WP) were measured. A principal component analysis (PCA) was used to describe the relationships involving the variables that accounted for data variance. *A. halimus* was shown to be the species most resilient to salinity, being able to maintain FW up to 300 mM, and RWC and WP up to 600 mM; it was followed by *C. album*. Compared to them, *A. vulgaris* and *S. komarovii* showed intermediate performances, achieving the highest FW (*A. vulgaris*) and GS (*S. komarovii)* under salinity. Lastly, *S. minor* and *A. absinthium* exhibited the most severe effects with a steep drop in GS and RWC. Lower WP values appeared to be associated with best halophyte performances under the highest salinity levels, i.e., 300 and 600 mM NaCl.

## 1. Introduction

The world population is expected to reach 9.7 billion people by 2050 and about 10.9 billion by 2100 [1]. To feed this growing population, food production shall increase by up to 70% by 2050 [2], although the progressive loss of fertile land due to soil degradation and urbanization will make this objective ever harder to achieve. This is further hampered by the rise in global warming, which is already increasing the occurrence and severity of drought events in formerly very productive land all over the world, increasing the pressure on high-quality water for irrigation [3], and highlighting the urgency of more resilient agricultural systems, especially in agricultural land suffering soil degradation and loss. On a global scale, salinity is one of the most severe factors of soil degradation, which affects over 100 countries and a land surface larger than 1 million hectares [4].

Natural soil salinization, known as primary salinization, occurs in arid and semi-arid climatic areas because of seawater intrusion, wind salt deposition, or parent material dissolution. Conversely, secondary salinization is induced by anthropogenic actions and is caused by the application of agrochemicals and by the use of low-quality irrigation water such as saline groundwater and wastewater [5]. Secondary salinization is expanding worldwide with a rate of 2 Mha year^−1^, posing a serious threat to agricultural productivity [6].

Unfortunately, even a minimum quantity of sodium chloride in irrigation water can cause severe yield losses in most agricultural crops. For example, the yield of bean, pepper, maize and potatoes was reported to decrease by 19%, 14%, 12% and 12%, respectively, at a salinity level of 2 dS m^−1^ [7]. There is thus the urgent need to identify alternative salt-resistant crops for farming and for the restoration of salt-affected areas in order to achieve the 2050 objective [8]. The state of the world’s plants and fungi 2020 report [9] of Kew Royal Botanic Gardens, drawn up by 210 researchers in 97 institutions across 42 countries, lists about 7000 edible plant species of which only 417 are included in the Food and Agriculture Organization’s (FAO) lists of major crops. Overlooked and underutilized plants may transform food production systems into more robust and ecologically sustainable ecosystems, through the cultivation of pre-adapted plants and the diversification of the spectrum of species used, while protecting biodiversity and essential ecosystem services.

Plants able to grow and thrive in saline environments are known as halophytes, and account for less than 2% of vascular plants. These species are able to grow in extreme environments characterized by high temperature, drought and salinity, such as coastal sand dunes, salt marshes and pans, and steppes where no crop would survive [10]. Halophytes are a source of fresh vegetables, medicinal and nutraceutical compounds, and provide multiple ecosystem services including soil protection from erosion and salinization, ornamental landscaping, wildlife support [11], and can mitigate the adverse effects of soil and water salinization on agricultural lands.

In this group of plants, *Artemisia absinthium* L., *Artemisia vulgaris* L., *Atriplex halimus* L., *Chenopodium album* L., *Salsola komarovii* Iljin, and *Sanguisorba minor* Scop. are six wild species with varying degrees of salt tolerance and of high potential agricultural value in the Mediterranean area.

*Artemisia* is a genus belonging to the Asteraceae family that is common in the temperate regions of the world. It includes around 500 species including perennial herbs and shrubs which were historically used in pharmaceutical and cosmetic preparations [12], thanks to their high content of active compounds such as essential oils, tannins, organic acids, carotenoids, ascorbic acid, and glycosides [13] with antioxidant, antiviral, and bactericidal activity [14,15]. *A. absinthium* has already been successfully used for salt- and heavy metal- phytoremediation [16,17,18,19]. *Artemisia* spp. are considered glycohalophytes, i.e., halfway between true halophytes and glycophytes, as they can control salt uptake by reducing root permeability to inorganic ions [20].

The two annual species *C. album* and *S. komarovii*, and the perennial *A. halimus* belong to the Amaranthaceae family (previously Chenopodiaceae) and occur in many world regions. *C. album* is an underexploited weed that, besides providing minerals, fibers, vitamins and essential fatty acids, was historically used for its medical properties as a blood purifier, diuretic, sedative, hepatoprotective, antiscorbutic laxative and as an anthelmintic drug [21,22]. Previous studies demonstrated the ability of *C. album* seeds to germinate in conditions of up to 400 mM soil salinity [23], to be able to select K^+^ over Na^+^ [24], and to produce osmo-compatible solutes and antioxidant enzymes that can prevent membrane lipids peroxidation under salt stress [25].

*Salsola komarovii,* also known as “agretti” in the Mediterranean region, was the main source of soda ash in past times [26]. Nowadays, it is sold as sea vegetable and salad crop at relatively high prices [27]. *Salsola komarovii* and *S. soda* are also used for soil salt and heavy-metal phytoremediaton [28,29,30], and in intercropping systems to increase the performance of glicophyte species [31,32,33]. Studies on the nutraceutical properties of *Salsola* spp. have also demonstrated their hypoglycemic effect [34,35] and their potential to contrast hypertension, constipation, inflammation, and Alzheimer’s disease [36,37].

*Atriplex halimus* is a shrub that is highly resistant to drought [38,39], salinity [40], and heavy-metal stress [41,42]. *Atriplex* species, indeed, are capable of excreting salts into special bladders located on the leaf [43]. A previous study documented increasing biomass under increasing salinity levels [44] in *Atriplex* spp., indicating that these plants can be key species in agro-ecosystems for saline soils cultivation and restoration, as well as for pasture and fodder production [45,46].

*Sanguisorba minor* is a perennial wild herb belonging to the Rosaceae family and mainly distributed in temperate areas of Europe. Known as salad burnet, it is a drought- and cold-tolerant species [47,48] that can also tolerate mild saline soils [49]. *S. minor* can be consumed as a fresh or boiled vegetable [50], or used for its medicinal and functional properties due to its high content of antioxidant bioactive compounds which provide digestive, antioxidant, astringent, carminative and diuretic properties [51,52].

Despite their multiple uses and a promising market, these species have a limited agricultural relevance at a local scale and are poorly known to farmers, as well as to agronomists and breeders.

The aim of this study is to increase the knowledge on how these salt-tolerant born species cope with salinity at the ecophysiological level and which saline conditions they prefer, can tolerate or are vulnerable to. Of these taxa, *Atriplex* and *Salsola* were already proposed as model organisms on which to focus the research in salt-adaptation in halophytes [53]. However, given the multitude of strategies evolved by halophytes, the comparison of species responses to salinity is more informative than studies concerning species-specific response of a single species and provide multiple reference systems that can serve as models to advance the research in salt-tolerance [53]. By comparing the ecophysiology of six halophytes in response to increasing salinity, this study establishes fundamental knowledge on which to build agronomic research in salt-tolerant crops and crop traits, and is the first step towards the resilient and sustainable agro-ecosystems that we all claim for the future.

## 2. Materials and Methods

### 2.1. Experimental Set Up

The experiment was carried out at the Department of Agricultural and Food Sciences (DISTAL), University of Bologna, Italy, and lasted 161 days, from 15 June to 23 November 2017. Commercial seeds of *Artemisia absinthium* L., *Artemisia vulgaris* L., *Atriplex halimus* L., *Chenopodium album* L., *Salsola komarovii* Iljin, and *Sanguisorba minor* Scop. were purchased from B&T World Seeds (Aigues-Vives, France) and Chiltern seeds (Wallingford, England) online shop. Seeds were surface-sterilized by immersion in a 3% sodium hypochlorite solution for 2 min, rinsed in deionized water for 5 min, and dried at room temperature. Seeds were then put in 9 cm Petri dishes on damp filter paper and placed into an incubator at 24 °C, 70–80% relative humidity, and 16/8 h light/dark period for 18 days. The filter paper was either dampened with distilled water (0 mM NaCl—control), or with four water solutions at increasing salinity (100, 200, 300, and 600 mM NaCl). The amount of salt added to water (TSS) to reach the salinity levels (EC_w_) was calculated according to Equation (1):

TSS (g_NaCl_ L^−1^ water) = EC_w_ (dS m^−1^) × 0.640 (1)

The germinated seeds (protruding radicles longer than 3 mm) were picked from the Petri dishes and moved into 2 L pots filled with peat moss growing media (26% organic carbon, pH_H_2_O_ = 7, and EC at 25 °C = 0.6 dS m^−1^). Germination was reduced under 300 mM NaCl and severely inhibited under 600 mM NaCl. Owing to this, seeds germinated in the range 0–300 mM NaCl were used for pots at various salinity levels according to this scheme: 0 mM NaCl (Petri dishes) → 0 and 100 mM NaCl (Pots); 100 mM NaCl (Petri dishes) → 200 mM NaCl (pot); 200 mM NaCl (Petri dishes) → 300 mM NaCl (pot); 300 mM NaCl (Petri dishes) → 600 mM NaCl (pot). Pots were transferred to a greenhouse.

The 6 species (HS) × 5 salinity levels (WS), totalling 30 combinations, were set up with three completely randomized replicates. This number of replicates is quite commonly adopted in pot experiments on this topic [24,54,55,56,57].

Ammonium nitrate (N, 26%) was added at 0.1 g pot^−1^ prior to seedling transplant; a second dose was supplied at mid-experiment by placing the granular fertilizer directly on the substrate surface prior to watering.

The pots were manually watered 2–3 times a week up to the end of the experiment. Around 9 L of saline solution was distributed per pot, corresponding to a NaCl amount of 57.6 g, 115.2 g, 172.8 g, and 345.6 g per pot in the four respective salinity levels. The maximum and minimum air temperature and relative humidity in the greenhouse were 28.47 ± 3.4 °C, 21.6 ± 4.6 °C, 71.0 ± 12%, and 49.8 ± 8.2%, respectively. Photosynthetically active radiation (PAR) of 200 μmol m^−2^ s^−1^ was provided by high pressure sodium lamps with a 16 h light and 8 h dark cycle.

### 2.2. Physiological Parameters Measurement

Leaf stomatal conductance (GS, mmol m^−2^ s^−1^) was measured at vegetative peak 28 days before harvest using a leaf porometer (AP4 Porometer, Delta-T Devices Ltd., Cambridge, England) equipped with a leaf chamber. Measurements were performed on the upper fully expanded leaf, on the middle portion of the blade between the midrib and the leaf margin.

Leaf water potential (WP, MPa) was assessed 21 days before harvest on a disk cut picked from the upper fully expanded leaf, using the WP4-C dewpoint potentiometer (METER Group, Pullman, WA, USA). Leaf cut were stored in sealed plastic cups and measurements were started within a short time of sampling [58]. Concurrently, leaf relative water content (RWC, %) was determined on the same leaf: a small disc of 2 cm diameter was cut from the leaf and weighed to determine fresh weight (FW). Then, it was put in a 15 mL vial with distilled water in the dark and after 24 h the turgid weight (TW) was measured. The sample was finally oven-dried at 105 °C for 24 h to assess the dry weight (DW). The RWC (%) was calculated according to Equation (2) [59]:

(2) $$\mathrm{RWC}=\frac{\mathrm{FW}\;-\;\mathrm{DW}\;}{\mathrm{TW}\;-\;\mathrm{DW}}\times 100$$

Since that *S. komarovii* has succulent threadlike leaves, a piece of branch from the upper part of the plant, with a surface area of about 2 cm^2^, was used for WP and RWC measurements.

At harvest, shoots were separated from roots and weighed to determine the plant fresh weight (FW, g plant^−1^). In *A. halimus* and *S. komarovii*, one of the three replicates treated with 600 mM and 200 mM WS, respectively, died before harvest. Therefore, we removed the incomplete FW data of these treatments from the dataset.

### 2.3. Statistical Analysis

The data of the four physiological traits (FW, GS, RWC and, WP) were submitted to analysis of variance (ANOVA) for the six species under control and the four saline treatments. We first analysed the overall effect of salinity in the 6 species vs. control, considering all the salinity levels together, i.e., the 12 salinity data collected for each trait for each species. This analysis, a one-way ANOVA, was meant to highlight the variation in salinity response and in tolerance extent among the six halophytes. Given the species-specific differences detected by this analysis, the measured traits, i.e., FW, GS, RWC and, WP, were normalized vs. their respective controls prior to the subsequent analyses (Equation (3)):

(3) $$Y_{r}=\frac{X_{i}-X_{c}}{X_{c}}\times 100$$

where *Y_r_* is the percentage difference of the parameter with respect to the control at the *i* salinity level, *X_i_* is the value of the parameter at *i* salinity level and *X_c_* is the mean value of the parameter in the control treatment.

Differences due to the specific salinity levels were analyzed by a two-way ANOVA using the species and the saline treatments as factors and testing their interaction. Since the interaction between the two factors resulted always significant at *p* ≤ 0.05 (except for RWC, *p* ≤ 0.1), meaning that the response to the saline treatment varied across the six species, the data were analyzed also with a one-way ANOVA within each species, to evaluate specific response to saline water treatment. The Tukey’s honest significant difference (HSD) post-hoc test at *p* ≤ 0.05 was used to indicate significant differences among species and/or treatments.

Two principal component analyses were carried out to summarize the performances of the six halophytes under control (Control PCA) and salt stress conditions (Control-normalized PCA). The principal components (PCs) were obtained from centered and scaled quantitative variables, through diagonalization of the correlation matrix and extraction of the associated eigenvectors and eigenvalues. In the Control PCA, the variables FW, GS, RWC, and WP were set as active quantitative variables, while the six halophyte species (HS) were used as supplementary categorical variables, i.e., variables that were not used in the computation of PCs. In the Control-normalized PCA, instead, the control-normalized FW, GS, RWC and, WP data were used as quantitative variables while the four saline water treatments (WS) and the six halophyte species (HS) were set as supplementary categorical variables.

A hierarchical clustering on the first two PCs (HCPC) was then realized both on control (Control HC) and saline treatments (Control-normalized HC) PCAs. The effect size (η^2^) was calculated for quantitative variables, i.e., plant physiological traits, to assess the proportion of the total variance associated with the extracted clusters that was explained by each trait. η^2^ was calculated as follows (Equation (4)):

(4) $$\eta^{2}=\frac{SS\;\;effect}{SS\;total}$$

where *SS effect* is the sum of squares for the quantitative variable effect, and *SS total* is the total sum of squares.

Clusters were then characterized using both quantitative and categorical variables through a test value (*v*-test). For quantitative variables, the cluster mean (*x_q_*) was compared with the overall mean *(x*), to ascertain if there was a significant difference within the cluster. The following quantity (Equation (5)) was calculated:

(5) $$u=\frac{x_{q}-x}{\sqrt{\frac{s^{2}}{n_{q}}\left(\frac{N-n_{q}}{N-1}\right)}}$$

where *n_q_* is the number of individuals in cluster *q*, *N* the total number of individuals, *s* the global standard deviation. The value of *u* was then compared to the corresponding quantile of the normal distribution; therefore, an absolute value greater than 1.96 implies a significant difference at *p* < 0.05, which in turn indicates the given variable as a characterizing one to discriminate the cluster. The sign indicates the direction of the deviation from the global mean.

For categorical variables, we aimed at identifying the category levels being over- or under-represented within the clusters. A χ^2^ test was performed between each categorical variable and the cluster variable. For the significant cases, the frequency *N_qj_* (number of individuals of the group q in the category level *j*) was represented in a hypergeometric distribution with the parameters *N, n_j_,* $\frac{n_{q}}{N}$ (where *n_j_* is the number of individuals that have taken the category *j*), and a *p*-value was calculated. The *p*-value was then transformed into the corresponding quantile value of the Gaussian distribution. Positive and negative signs mean over- and under- representation, respectively, of the referred category within the specific cluster.

All the statistical analyses were performed with the R 6.3.6 statistical software, using Car [60] and Emmeans [61] packages for the analysis of variance and post-hoc test, and the FactoMineR package for principal component analysis and hierarchical clustering on principal components [62]. Charts were created with the ggplot2 [63] R packages.

## 3. Results

### 3.1. Control versus Saline Water Treatments

**Figure 1:**
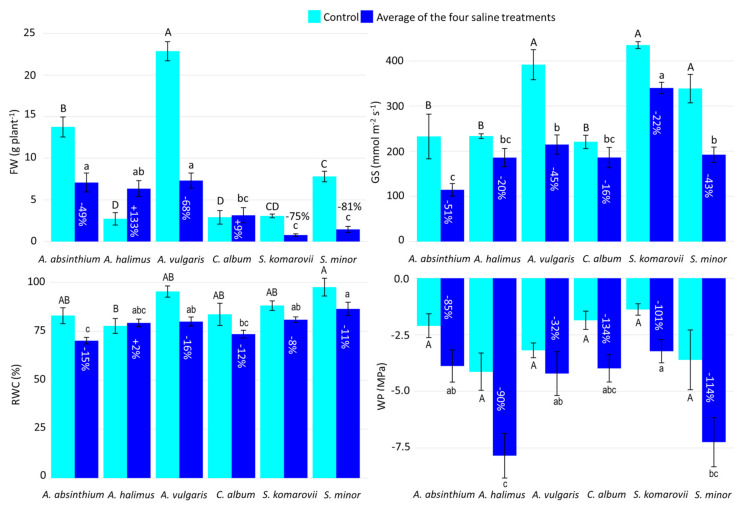
Fresh weight (FW), stomatal conductance (GS), leaf relative water content (RWC), and leaf water potential (WP) of the six halophyte species under control (cyan histograms), and the four saline treatments combined (blue histograms). The percent difference between control and the combined saline treatment is reported for each species as % in the saline treatment column. Uppercase and lowercase letters indicate statistical differences (*p* ≤ 0.05) among the six species in controls and average saline treatment respectively. Vertical bars indicate ± one standard error.

The performance of the six halophyte species under saline conditions (100–600 mM NaCl) vs. control are displayed in Figure 1. Under control conditions, *A. vulgaris*, *A. absinthium* and *S. minor* showed, in decreasing order, high FW, while the other three species had a similar, low FW. Under salinity, *A. vulgaris*, *A. absinthium* and *A. halimus* were top ranked with respect to FW, which means that the first two species plummeted with respect to the control (−68% and −49%), while the third species soared (+133%). They were followed by *C. album*, whose FW did not substantially vary from the control, while *S. komarovii* and *S. minor* were bottom ranked and showed the strongest drops vs. control conditions (−75% and −81%, respectively). The lack of the 600 mM and 200 mM WS data in *A. halimus* and *S. komarovii*, respectively, might have increased and decreased the FW mean value under salinity, in the two respective cases.

*A. vulgaris, S. komarovii* and *S. minor* showed the highest stomatal conductance values under control conditions, followed by the three remaining species having similar lower GS values. Under salinity, *S. komarovii* had the highest GS, followed by the group of *A. vulgaris*, *S. minor, C. album* and *A. halimus*. The lowest GS was shown by *A. absinthium*, resulting in the strongest drop from the control (−51%).

Relative water content outlined narrow, yet significant, differences under control conditions: *S. minor* had the highest RWC, while *A. halimus* had the lowest RWC. Under salinity, *S. minor* remained top ranked, followed by *S. komarovii*, *A. vulgaris* and *A. halimus*. Lastly, *C. album* and *A. absinthium* were the two bottom-ranked species. In the case of *A. halimus,* no RWC difference was observed, in practice, between control and salinity, while in the other species the decrease due to salinity ranged between 8% (*S. komarovii*) and 16% (*A. vulgaris*).

Water potential did not significantly vary under control conditions. Under saline treatments, *A. halimus* and *S. minor* dropped to significantly lower WP levels than *S. komarovii*, while the other three species were intermediate. The relative decreases vs. control conditions ranged from a minimum of 32% (*A. vulgaris*) to a maximum of 134% (*C. album*).

### 3.2. Normalized Water Saline Treatments

To better compare the performances of the halophyte species, the values of each physiological trait were normalized with respect to the mean control value. The two-way ANOVA (Table 1) of normalized data showed a significant HS × WS interaction for FW, GS, WP and, to a less extent (*p* ≤ 0.1), RWC, indicating a different effect of saline treatments within species.

**Figure 2:**
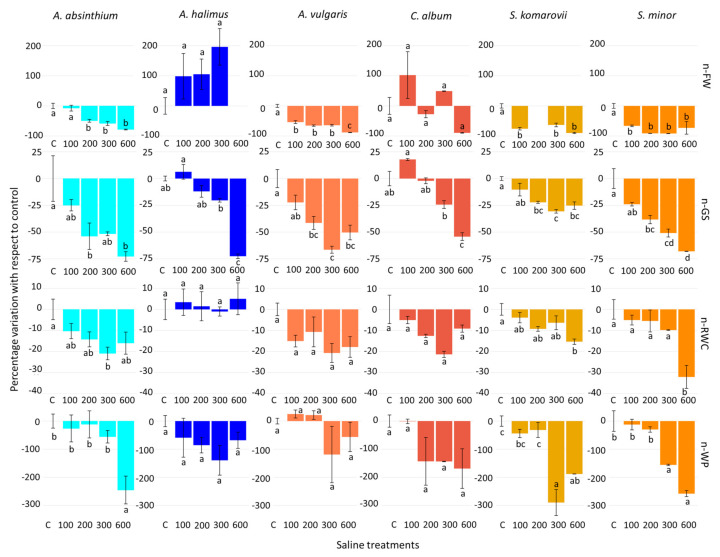
Control-normalized percent differences in fresh weight (n-FW), stomatal conductance (n-GS), leaf relative water content (n-RWC), and leaf water potential (n-WP) within species due to the different saline treatments experimented in the study. Different letters indicate significant differences at *p* ≤ 0.05. Vertical bars indicate ± one standard error. In *A. halimus* and *S. komarovii*, one n-FW level is missing due to dead plants at harvest.

Accordingly, a one-way ANOVA (Table 2) was performed to assess the effects of salinity within each species for the above-mentioned physiological traits. Salinity determined significant variations in all traits and species, with the exception of n-FW, n-RWC and n-WP in *A. halimus*, n-RWC in *C. album*, and n-WP in *A. vulgaris*. The effects across increasing salinity levels are displayed in Figure 2.

Saline treatments determined significant n-FW decreases in all species except *A. halimus* and *C. album.* Higher FW values in salt treatments than controls may indicate that lack of salt, rather than salinity, is a limiting factor in *A. halimus* and *C. album*. However, for *A. halimus* we cannot assess the effect of the 600 mM NaCl treatment, owing to missing data. Only in *A. vulgaris* the highest salinity level, 600 mM NaCl, had a significant stronger negative effect than the lower saline levels, whereas in *A. absinthium, S. komarovii* and *S. minor* there was no significant effect beyond 100 mM NaCl (*S. komarovii* and *S. minor*) or 200 mM NaCl (*A. absinthium*) (Figure 2). Hence, for *S. komarovii* the lack of the 200 mM NaCl treatment data appears less critical than the lack of 600 mM for *A. halimus*, because higher salinity levels (300 and 600 mM NaCl) did not exhibit a different FW response compared to the 100 mM NaCl treatment.

Salinity determined significant n-GS decreases in all species, and the effect was quite proportional to the salinity levels (Figure 2). Salinity, as well, induced significant n-RWC decreases in *A. absinthium, S. komarovii* and *S. minor*. The effect was consistent only in the highest salinity treatment (600 mM NaCl) in *S. Komarovii* and *S. minor* (Figure 2). Salinity also determined significant n-WP decreases in these three species; the effect was significant from 300 mM NaCl (*S. komarovii* and *S. minor*) or with 600 mM NaCl (*A. absinthium*) (Figure 2). *A. vulgaris, A. halimus*, and *C. album* did not show RWC and WP changes across salinity levels.

### 3.3. Results of the Multivariate Analysis

Two PCAs were performed to summarize with a multivariate approach the performance of the six halophyte species under control (Control PCA) and salt stress treatments (Control-normalized PCA).

**Figure 3:**
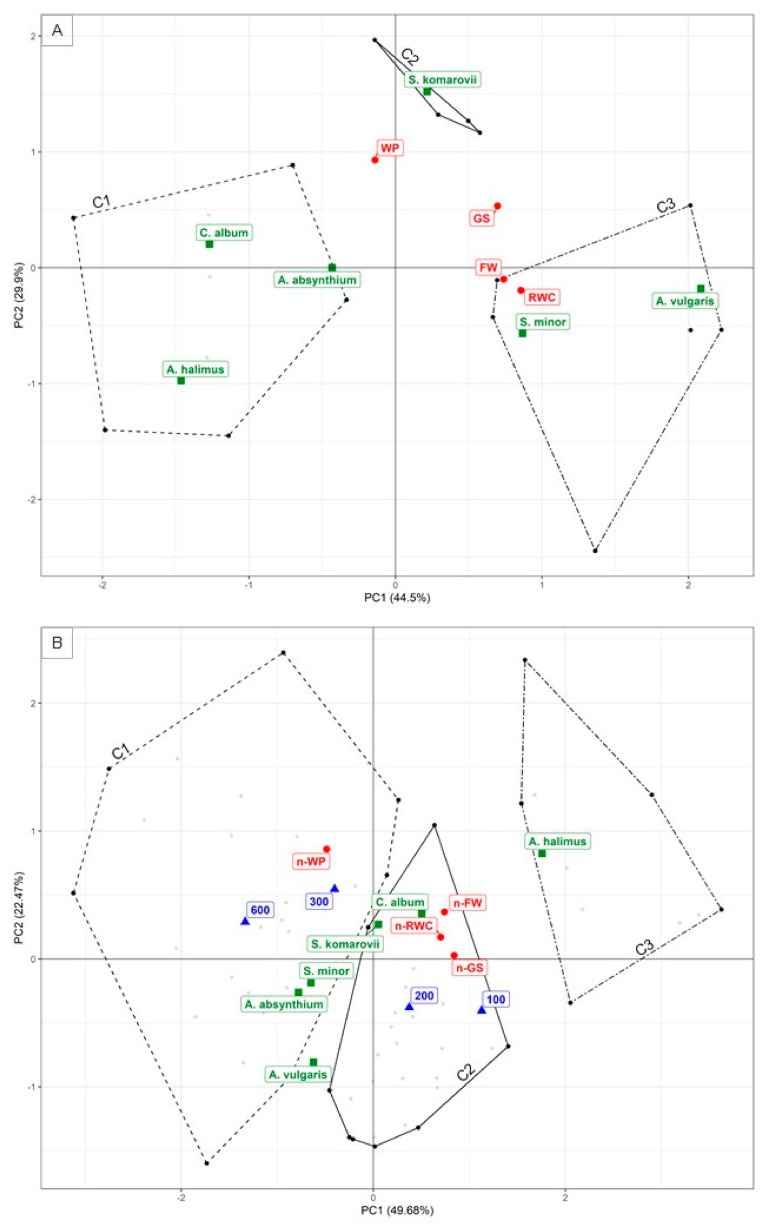
(**A**) Control and (**B**) Control-normalized principal component analysis (PCA) biplot of variables. Green points show the barycenters of the halophytes species (HS), blue point show the barycenters of the saline treatments (WS), red points show the quantitative variables, i.e., fresh weight (FW), stomatal conductance (GS), leaf relative water content (RWC), and leaf water potential (WP). The lowercase n indicates control-normalized data. The polygons show the three extracted clusters in the first two PCs space. Grey dots represent the individuals of the two categorical variables, i.e., WS and HS. C1, C2 and C3 = Cluster 1, 2, and 3.

The first two PCs (eigenvalues are reported in Table S1 of the Supplementary Materials) were used for PCA interpretation, explaining 74.4% and 72.2% of the total variance in the respective Control PCA and Control-normalized PCA (Figure 3). The correlation coefficients were calculated between the PCs and each quantitative (the four physiological traits) and qualitative, i.e., categorical (the six halophytes species and the four saline treatment) variables (PCAs correlation circles in Figure S1 of the Supplementary Materials). The associated *p*-values were computed to rank the variables according to their relevance (Table S2 of the Supplementary Materials).

Under both control (Control PCA) and salt stress treatments (Control-normalize PCA), FW, GS and RWC were positively correlated with PC1, suggesting a high degree of multicollinearity among these parameters (red barycenters in Figure 3). WP, instead, resulted to be positively correlated with PC2 in both PCAs, and negatively correlated with PC1 under saline treatments (Table S2). WP, indeed, was located on the negative side of PC1 (Figure 3), at the opposite side of FW, GS, and RWC which are placed at positive values. This is in agreement with the fact that a reduction in WP (more negative values) should contrast FW, GS and RWC reductions caused by the NaCl related osmotic stress and ion toxicity.

The position of the barycenters of the six species in the two PCs changed drastically from control to saline treatments (Figure 3). In the Control PCA, the PC1 had negative loadings for *A. halimus* and *C. album*, and positive loadings for *A. vulgaris* and *S. minor* (green barycenters in Figure 3A). Conversely, in the Control-normalized PCA, the PC1 had negative loadings for *A. vulgaris* and *S. minor*, and positive loadings for *A. halimus* and *C. album* (green barycenters in Figure 3B). This suggests that the last two species were the best performing under salt stress conditions (no substantial FW, GS and RWC drop) although they resulted in being the worst performing under control conditions (lowest FW, GS, and RWC values). The barycenters of *S. komarovii* and *A. absinthium* in the PCA biplot did not vary substantially along PC1 due to saline treatments. *S. komarovii* was still located on the positive side of the PC1, as it showed high-intermediate ranking both under control (highest GS) and salinity conditions (highest GS and WP)*. A. absinthium*, as well, remained located on the negative side of the PC1, showing an intermediate performance under control and a low-intermediate performance under salinity (lowest GS and RWC). *A. absinthium* was likely located on the negative side of the PC1 because, despite the restrained FW drop, this species was the last ranked for RWC and GS under salinity (Figure 1).

Under salt treatments (Control-normalized PCA), hence, the HS barycenters (green square in Figure 3B) followed a linear trend, with the best performing species (*A. halimus* and *C. album*) located in the upper-right quadrant, in the same direction as RWC, FW, and GS (red dot in Figure 3B), opposite to the worst performing species placed in the bottom-left quadrant. The barycenters of the water saline treatments (WS) (blue triangles in Figure 3B), instead, followed an inverse gradient, crossing the HS linear trend, with the two lowest WS treatments (100 and 200 mM NaCl) in the right-bottom quadrant, and the two highest WS treatments (300 and 600 mM NaCl) in the upper-left quadrant, at the opposite side of the FW, RWC and GS quantitative variables. Hence, the best performing species (*A. halimus*, *C. album* and *S. komarovii*) and the two highest saline treatments (300 and 600 mM NaCl) resulted in being located on the positive side of the PC2. As already mentioned, the PC2 was strongly correlated with the WP (Table S2), thereby suggesting that *A, halimus*, *C album* and *S. komarovii* respond to high salinity by lowering WP, i.e., by osmotic adjustment.

### 3.4. Cluster Analysis

The hierarchical clustering (HC) extracted three clusters in the first two PCs space, both for control (Control HC) and salt stress treatments (Control-normalized HC) (Figure 3A,B). Effect size ($\eta^{2}$) and χ^2^ test values, expressing the link between the cluster variable and the respective quantitative and categorical variables, are reported in Tables S2 and S3, respectively, of the Supplementary Materials.

In the Control HC, the first cluster (C1) was characterized by average RWC and GS values lower than the overall mean (80.25 vs. 87.55% and 220 vs. 308.67 mmol m^−2^ s^−1^, respectively) (Table S5). None of the categorical variables significantly characterized this cluster, although 100% of *A. halimus* and *C. album* individuals were linked to it at *p* = 0.068 ^(+)^, (Table S6). The second cluster (C2), instead, was characterized by GS mean values higher than the overall mean (425 vs. 308.67 mmol m^−2^ s^−1^), in exchange for WP mean values lower than the overall mean (−1.39 MPa vs. −2.71 MPa) (Table S7). This cluster was mainly represented by *S. komarovii* (100% of the individuals) (Table S8). The third cluster (C3), finally, was characterized by FW and RWC mean values higher than the overall mean (16.5 vs. 8.86 g plant^−1^ and 96.54 vs. 87.55%, respectively) (Table S9). *A. vulgaris* was primarily present in this cluster (100% of the individuals) (Table S10).

Under salt treatments (Control-normalized HC), the WS categorical variables (i.e., water saline treatments) characterized the clusters better than HS categorical variables (i.e., halophytes species). The C1 mainly contained WP, FW, RWC and GS mean values lower than the overall mean (−170.05% vs. −90.54%, −62.40% vs. −27.01%, −16.22% vs. −10.28%, and −50.86% vs. −34.10%, respectively), (Table S4). This cluster, indeed, was described by the 300 mM and 600 mM NaCl WS levels (82.35% and 85.71% of the respective individuals), whose frequency in C1 was much higher than their frequency in the complete dataset (50% vs. 26.15% and 42.86% vs. 21.54%, respectively), (Table S6). Hence, C1 may be indicated as the cluster describing the most severe effects associated with the highest salinity levels.

Cluster C2 was characterized by FW and WP mean values lower than the overall mean (−53.77% vs. −27.02% and 17.27% vs. 90.54%, respectively) (Table S7), and mainly grouped the two WS levels 100 mM and 200 mM NaCl (76% and 71% of the respective individuals), whose frequency in C2 was much higher than in the whole dataset (48.15% and 44.44% vs. 26.15% global mean for the two respective categories) (Table S8). C2, hence, may be indicated as the cluster describing the intermediate effects connected to moderate salinity levels.

Cluster C3, finally, was characterized by FW, GS, and RWC mean values higher than the overall mean (144.25% vs. −27.01%, −3.54 vs. −34.10%, and 0.39% vs. −10.28%) (Table S9). This was the only cluster that was meaningfully described by a HS category, i.e., the *A. halimus* species (with 72.73% of the individuals), whose frequency in C3 was 80% against 16.92% in the whole dataset (Table S10). The other species did not reveal any clear pattern according to the three clusters. Hence, C3 may be summarized as the cluster representing the most effective physiological reactions involved in salt tolerance.

## 4. Discussion

Halophytes are a small group of plants naturally adapted to survive salinity in areas where almost all other terrestrial species cannot grow. This work compares some physiological traits of six common halophytes in the Mediterranean area whose cultivation may promote the recovery and reuse of salt-degraded lands. Additionally, they may provide an alternative to, or can diversify, conventional crops [64], and reduce agricultural pressure on good quality land and water resources.

The six species showed different strategies to cope with salinity associated with their specific functional and life history traits. *Artemisia* spp. and *A. halimus* are for example perennial shrubs growing up to 1–2 m height, *C. album* is an annual herbaceous plant 1 to 1.5 m tall, *S. minor* is a perennial rhizomatous forb with erect stems 2 to 70 cm tall, while *S. komarovii* is an annual species growing up to 30 cm, with long needle-like succulent leaves. The study showed also species-specific salinity thresholds beyond which stress became evident, in different ways, making this dataset an important base of knowledge for the research in salt-tolerance in plants and for the selection of species for future crops.

*A. vulgaris* and *A. halimus* showed, respectively, the highest and lowest FW (∼14 and 3 g plant^−1^, respectively) under control. *A. vulgaris* and *A. halimus* have different life history traits; indeed, *A. vulgaris* is a fast growing species, while *A. halimus* has been classified as a medium growth rate species [65]. This may explain why the former produced a higher FW over the same period. However, while *A. vulgaris* FW dropped by 68% under water salinity, *A. halimus* FW increased up to 300 mM NaCl (+133%), although not significantly, in accordance with previous studies that showed no reduction in *A. halimus* biomass up to 600 mM NaCl [66,67]. However, other studies showed a significant decrease in *A. halimus* biomass with salinity above 200 mM NaCl [38,46]. Like *A. halimus*, *C. album* growth was not affected by salinity. This is in contrast with the results of Rasouli et al. [68], who found that FW dropped already at 100 mM NaCl in *C. album*; conversely, it is in agreement with the findings of Ivanova et al. [69], who did not record biomass variations up to 200 mM NaCl.

By contrast with the other four species, GS did not decrease in *A. halimus* and *C. album* up to 200 mM NaCl, but on the contrary increased at 100 mM NaCl (Figure 2), although the GS values of these two species were among the lowest under control treatment. In contrast to our results, Pérez-Romer et al. [40] did not observe any GS change in *A. halimus* up to 513 mM NaCl, whereas Rasouli et al. [68] observed a significant GS drop in *C. album* already at 100 mM NaCl.

Halophytes exposed to increasing salinity may activate a partial stomatal closure as a mechanism to limit both transpiration and transport of salts to the leaves [70]. A decline in GS, however, is usually associated with a reduced diffusion of CO_2_ leading to a lower carboxylation efficiency. Rasouli et al. [68], however, observed that the CO_2_ assimilation rate in salt-grown halophytes is largely unrelated to stomatal conductance since they were able to sustain FW notwithstanding the limited GS. This was attributed to a faster, thus more efficient, stomatal opening and closure regulation and to a faster RuBisCo carboxylation rates under salinity.

This mechanism may explain the steadiness in FW shown by *A. halimus* and *C. album* at increasing salinity, despite the GS drop above 200 mM NaCl.

The two *Artemisia* species reacted differently to salinity, as *A. absinthium* showed a stronger decline in GS (−51%) than that shown by *A. vulgaris* (−45%) (Figure 1), resulting to be the last ranked species for this parameter. Under salinity, indeed, GS value dropped below 200 mmol m^−2^ s^−1^ in *A. absinthium* (Figure 1), similarly to what observed by Aftab et al. [71] in *Artemisia annua* under 200 mM NaCl.

Another trait characterizing *A. halimus* and *C. album*, and shared also by *A. vulgaris,* was RWC steadiness under salinity (Figure 1). Similarly, Paulino et al. [72] and De Araújo et al. [73] observed, no RWC decrease up to 600 mM NaCl in *Atriplex nummularia* despite decreased transpiration rates. Ivanova et al. [69], as well, remarked the ability of *C. album* to maintain unaltered water status up to 350 mM NaCl water salinity, while Lu et al. [74] observed a reduction in its water content already at 300 mM NaCl. Likewise, *S. minor* showed a steadiness in RWC up to 300 mM NaCl, but then experienced a drastic RWC drop with the highest NaCl level. This is in contrast with the findings of Shariat et al. [75] who observed a *S. minor* RWC reduction at already 100 mM NaCl. *A. absinthium,* instead, reached a significant RWC drop already at 300 mM NaCl, contrary to the findings of Sharifivash et al. [76], who observed a first significant reduction in RWC at 150 mM NaCl in *A. absinthium.*

The ability to keep unaltered leaf RWC under salt stress suggests that *A. halimus*, *C. album* and *A. vulgaris* evolved adequate mechanisms to ensure sufficient water uptake in saline soils. According to Ben Amor et al. [77], salt tolerance of dicotyledonous halophytes is strongly related to their ability to accumulate high levels of osmolytes in their tissues and, thus, to adjust osmotically by lowering the osmotic potential in the cytoplasm. A decrease in WP, however, could also originate from tissue dehydration, although this does not seem to be the case for *A. halimus*, *C. album*, and *A. vulgaris* that did not change in RWC with saline treatments. The osmotic adjustment (OA) allows root water uptake and leaf turgor to be maintained under conditions of low soil water potential [78]. When turgor is maintained, all the turgor-dependent processes, i.e., stomatal conductance, assimilation rate and cellular wall expansion, can be maintained, albeit to a reduced rate due to the OA-associated metabolic costs [79]. This mechanism is fully supported by the results of the PCAs (Figure 3), that placed WP loadings in an opposite distinct position from the other physiological traits, supporting the hypothesis that the capability to sustain RWC, FW and GS depends on the magnitude of WP lowering.

Hence, although *A. halimus, C. album*, and *A. vulgaris* did not show a significant WP decrease with salinity, their similar and low WP values may have favoured root water uptake and in turn RWC maintenance (Figure 1 and Figure 2*).*

*S. komarovii* showed the highest WP under control and this may be due to its succulent habit. Succulent plants, indeed, have larger vacuole where they store water and this likely prevents the development of low water potentials in their photosynthetic tissues [80]. Succulence usually tends to increase under saline conditions [66] in such a way that the increasing vacuole volume may serve to compartmentalize and dilute salts and help handle temporary imbalances due to NaCl introduction into the plant [64]. Despite this, however, *S. komarovii* showed a WP decrease under 300 mM NaCl and was not able to preserve its RWC over this salinity threshold.

Overall, the discrepancy sometimes observed in the response to salinity across the six species may be attributed to intraspecific variation in populations originating from different habitats, as well as to different growth conditions in the cited experiments. The latter circumstance supports the need for harmonization in the protocol of salinity experiments, as a premise for more consistent results.

## 5. Conclusions

Our results indicate that *A. halimus* and *C. album* are the best adapted species to salinity, followed by the group of *S. komarovii* and *A. vulgaris*, whereas *S. minor* and *A. absinthium* emerged as the least capable to adapt to increasing salinity levels.

*A. halimus* had low-intermediate FW, RWC and WP values under control treatment, and these traits remained almost stable under saline treatments. As a matter of fact, *A. halimus*, a C4 species, constituted a clearly distinctive cluster under salinity with respect to the other species, all at C3 photosynthetic pathway, and displayed the most consistent tolerance to salinity. *C. album* was also resilient (no FW, RWC and WP reduction), but less tolerant to salinity than *A. halimus*, as *C. album* data were split between the first and third cluster. These two species, however, appeared to be stimulated by a weakly saline environment, as their FW and GS increased slightly. *S. komarovii* was characterized by the highest GS values both under control and the highest salinity level, although with a significant drop in FW, RWC and WP. *A. vulgaris* showed the highest FW under control, but its FW was halved under salinity; however, its RWC and WP were not affected by salinity. *S minor* showed the highest RWC under control and was able to preserve it up to 300 mM NaCl; however, it showed a severe FW, GS, and WP drop. Finally, *A. absinthium* had one of the highest FW both under control and salinity, but suffered the highest GS and RWC drop already at 100 mM NaCl.

The physiological parameters addressed in this study are essential to assess salt-tolerance and appreciate differences in salt-adaptation among species. However, our study is far from being exhaustive as it concerns the processes associated with salinity response in halophytes. Although incomplete, this study opens several research windows and perspectives concerning (a) the actual production and/or accumulation of organic and inorganic osmolytes, in order to evaluate their contribution to the plant’s osmoregulation; (2) the effective changes in stomata shape and density with salinity, and the overall salinity effect on the photosynthetic capacity; and finally, (3) the cellular wall elasticity changes, which play an important role in a plant’s ability to regulate its water relations.

## Figures and Tables

**Table 1:** F values and statistical significance in the two-way analysis of variance (halophyte species × water salinity levels) carried out on the control-normalized values of the four measured physiological traits: fresh weight (n-FW), stomatal conductance (n-GS), leaf relative water content (n-RWC), and leaf water potential (n-WP).

Source	dF	n-FW	n-GS	n-RWC	n-WP
HS	5	21.74 **	15.67 **	7.36 **	2.18 ^(+)^
WS	4	7.56 **	61.88 **	9.99 **	21.07 **
HS × WS	20	3.91 **	3.18 **	1.67 ^(+)^	1.91 *

WS = Water salinity; HS = Halophyte species. Significance codes: ^(+)^, * and ** mean significant at *p* ≤ 0.1, *p* ≤ 0.05 and *p* ≤ 0.01, respectively.

**Table 2:** F values and statistical significance in the one-way analysis of variance carried out on the control-normalized percent values of the four measured physiological traits: fresh weight (n-FW), stomatal conductance (n-GS), leaf relative water content (n-RWC), and leaf water potential (n-WP).

Halophyte Species	dF	n-FW	n-GS	n-RWC	n-WP
*A. absinthium*	4	24.05 **	6.14 **	3.60 *	6.28 **
*A. vulgaris*	4	90.46 **	15.60 **	2.93 ^(+)^	1.41 ^ns^
*A. halimus*	4	2.02 ^ns^	38.19 **	0.17 ^ns^	1.26 ^ns^
*C. album*	4	3.76 ^(+)^	27.62 **	3.10 ^ns^	3.48 ^(+)^
*S. komarovii*	4	46.41 **	14.60 **	4.64 *	19.40 **
*S. minor*	4	29.28 **	22.15 **	8.11 **	24.73 **

Significance codes: ^ns^, ^(+)^, * and ** mean non-significant and significant at *p* ≤ 0.1, *p* ≤ 0.05 and *p* ≤ 0.01, respectively.

## Data Availability

Not applicable.

## Supplementary Materials

The following are available online at https://www.mdpi.com/2223-7747/10/2/309/s1, Figure S1: Correlation circle of active quantitative variables in (A) Control PCA and (B) Control-normalized PCA on the four physiological traits: fresh weight (FW), stomatal conductance (GS), leaf relative water content (RWC), and leaf water potential (WP); the lowercase n indicates control-normalised data. The amount of variation explained by each principal component (Dim) is indicated in brackets, Table S1. Eigen analysis of the correlation matrix in Control and Control-Normalized PCA, Table S2. Correlation coefficients between quantitative and categorical variables, and the first two PCs in Control PCA and Control-normalized PCA. FW = fresh weight, GS = stomatal conductance, RWC = leaf relative water content, and WP = leaf water potential; the lowercase n indicates control-normalised data. The Control PCs were computed using 18 input data, while the Control-normalized PCs were computed using 65 input data, Table S3. $\eta^{2}$ values calculated for each quantitative variable, indicating the amount of explained between-clusters variance; only significant results are shown, Table S4. *p* values of the $\chi^{2}$ tests performed between the categorical variables and the extracted clusters, Table S5. *v*-test results for cluster I quantitative variables; only significant results are shown. A positive or negative test statistic indicates a cluster mean significantly higher or lower, respectively, than the global mean. Cluster and global mean and standard deviation are also reported. Variables are ordered by value of *v*-test. Table S6. *v*-test results for Cluster I categorical variables.; only significant results are shown. Within-cluster (Mod/Cla) and across-cluster (Cla/Mod) distributions, and global cluster mean of categorical variables are also reported. Variables are ordered by value of *v*-test, Table S7. *v*-test results for Cluster II quantitative variables; only significant results are shown. A positive or negative test statistic indicates a cluster mean significantly higher or lower, respectively, than the global mean. Cluster and global mean and standard deviation are also reported. Variables are ordered by value of v-test, Table S8. *v*-test results for Cluster II categorical variables; only significant results are shown. Within-cluster (Mod/Cla) and across-cluster (Cla/Mod) distributions, and global cluster mean of categorical variables are also reported. Variables are ordered by value of *v*-test, Table S9. *v*-test results for Cluster III quantitative variables; only significant results are shown. A positive or negative test statistic indicates a cluster mean significantly higher or lower, respectively, than the global mean. Cluster and global mean and standard deviation are also reported. Variables are ordered by value of *v*-test, Table S10. *v*-test results for Cluster III categorical variables; only significant results are shown. Within-cluster (Mod/Cla), and across-cluster (Cla/Mod) distributions, and global cluster mean of categorical variables are also reported. Variables are ordered by value of *v*-test.
