# Supplementary material for: Physiological Adaptation to Water Salinity in Six Wild Halophytes Suitable for Mediterranean Agriculture
Source: Plants (Basel). 2021 Feb 5;10(2):309. doi: 10.3390/plants10020309 (PMC7914791; doi:10.3390/plants10020309)
Supplement: Supplementary file 1 [file plants-10-00309-s001.pdf]

Table S1. Eigen analysis of the correlation matrix in Control and Control-Normalized principal component analysis (PCA)

		Eigenvalue	Percentage of Variance	Cumulative Percentage of Variance
**Control PCA**	**PC1**	1.78	44.54	44.54
	**PC2**	1.20	29.91	74.45
**Control-normalized PCA**	**PC1**	1.99	49.65	49.65
	**PC2**	0.90	22.53	72.17

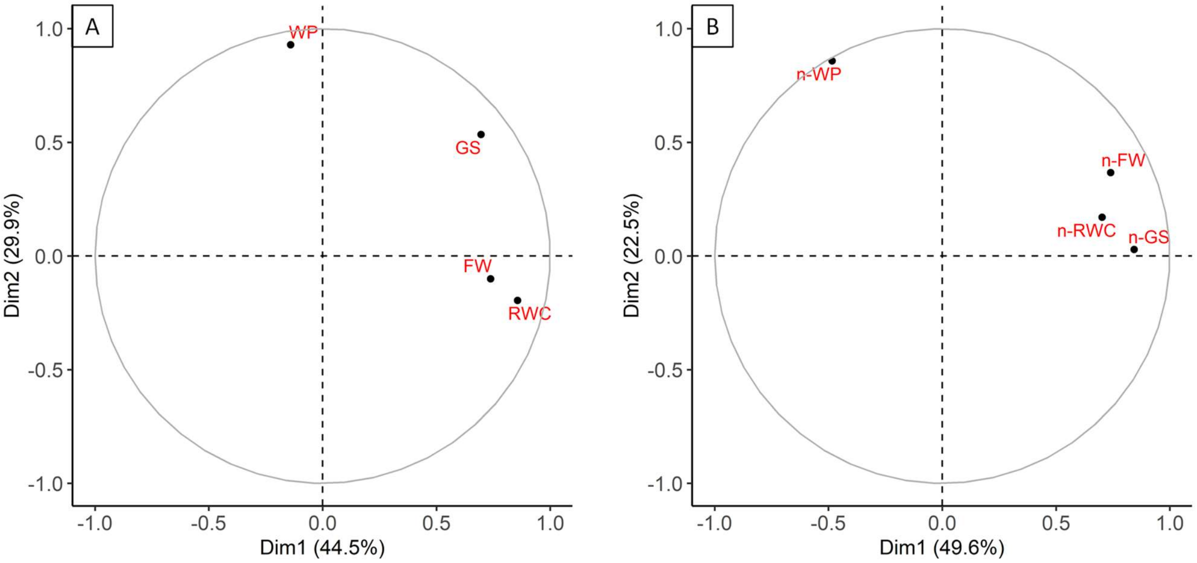

Figure S1: Correlation circle of active quantitative variables in (**A**) Control PCA and (**B**) Control-normalized PCA on the four physiological traits: fresh weight (FW), stomatal conductance (GS), leaf relative water content (RWC), and leaf water potential (WP); the lowercase n indicates control-normalised data. The amount of variation explained by each principal component (Dim) is indicated in brackets.

Table S2. Correlation coefficients between quantitative and categorical variables, and the first two PCs in Control PCA and Control-normalized PCA. FW = fresh weight, GS = stomatal conductance, RWC = leaf relative water content, and WP = leaf water potential; the lowercase n indicates control-normalised data. The Control PCs were computed using 18 input data, while the Control-normalized PCs were computed using 65 input data.

Variable	Control PCA		Control-normalized PCA	
	**PC1**	**PC2**	**PC1**	**PC2**
Quantitative variables				
(n-)FW	0.74**	-0.10^{ns}	0.74**	0.37**
(n-)GS	0.70**	0.53*	0.85**	0.05^{ns}
(n-)RWC	0.86**	-0.20^{ns}	0.70**	0.15^{ns}
(n-)WP	-0.14^{ns}	0.92**	−0.48**	0.86**
Categorical variables				
WS			0.41**	0.20**
HS	0.85**	0.51^{ns}	0.40**	0.31**

WS = Water salinity. HS = Halophyte species. Significance codes: ^{ns}, * and ** mean non-significant and significant at $p \leq 0.05$ and $p \leq 0.01$, respectively.

Table S3. η^2 values calculated for each quantitative variable, indicating the amount of explained between-clusters variance; only significant results are shown

	Control HC	Control-based HC
**n-FW**	0.5222	0.7200
**n-GS**	0.8036	0.4725
**n-WP**	-	0.4586
**n-RWC**	0.6241	0.3495

Table S4. P values of the χ^2 tests performed between the categorical variables and the extracted clusters.

	Control HC		Control-based HC	
**Variable**	**Df**	**p-value**	**Df**	**p-value**
**SW**	-	-	6	< 0.001
**HI**	10	0.0022	10	< 0.001

Table S5. v-test results for Cluster I quantitative variables; only significant results are shown. A positive or negative test statistic indicates a cluster mean significantly higher or lower, respectively, than the global mean. Cluster and global mean and standard deviation are also reported. Variables are ordered by value of v-test.

	Variable	v-test	Cluster mean	Global mean	Cluster SD	Global SD
**Control- HC**	**n-RWC**	−2.98	80.25	87.55	6.51	9.03
	**n-GS**	−3.48	220.00	308.67	36.81	93.69
**Control-based HC**	**n-WP**	5.27	170.05	90.54	93.71	104.91
	**n-FW**	−2.87	−62.40	−27.01	36.80	85.68
	**n-RWC**	−4.15	−16.22	−10.28	8.48	9.97
	**n-GS**	−4.79	−50.86	−34.10	19.10	24.35

Table S6. v-test results for Cluster I categorical variables.; only significant results are shown. Within-cluster (Mod/Cla) and across-cluster (Cla/Mod) distributions, and global cluster mean of categorical variables are also reported. Variables are ordered by value of v-test.

	Variable	Cla/Mod	Mod/Cla	Global mean	v.test
**Control- HC**	**NULL**				
**Control-based HC**	**WS 300**	82.35	50.00	26.15	3.73
	**WS 600**	85.71	42.86	21.54	3.55
	**HS *A. halimus***	9.09	3.57	16.92	−2.49
	**WS 200**	11.76	7.14	26.15	−3.05
	**WS 100**	0.00	0.00	26.15	−4.45

Table S7. v-test results for Cluster II quantitative variables; only significant results are shown. A positive or negative test statistic indicates a cluster mean significantly higher or lower, respectively, than the global mean. Cluster and global mean and standard deviation are also reported. Variables are ordered by value of v-test

	Variable	v-test	Cluster mean	Global mean	Cluster SD	Global SD
**Control- HC**	**n-GS**	2.74	425.00	308.67	19.69	93.69
	**n-WP**	2.06	−1.39	−2.71	0.31	1.41
**Control-based HC**	**n-FW**	−2.09	−53.77	−27.02	26.16	85.69
	**n-WP**	−4.71	17.27	90.54	55.30	104.91

Table S8. v-test results for Cluster II categorical variables; only significant results are shown. Within-cluster (Mod/Cla) and across-cluster (Cla/Mod) distributions, and overall cluster mean of categorical variables are also reported. Variables are ordered by value of v-test.

	Variable	Cla/Mod	Mod/Cla	Global mean	v.test

**Control- HC**	**HS *S. komarovii***	100.00	75.00	16.67	2.81
**Control-based HC**	**WS 100**	76.47	48.15	26.15	3.29
	**WS 200**	70.59	44.44	26.15	2.72
	**WS 600**	14.29	7.41	21.54	−2.31
	**WS 300**	0.00	0.00	26.15	−4.32

Table S9. v-test results for Cluster III quantitative variables; only significant results are shown. A positive or negative test statistic indicates a cluster mean significantly higher or lower, respectively, than the global mean. Cluster and global mean and standard deviation are also reported. Variables are ordered by value of v-test.

	**Variable**	**v-test**	**Cluster mean**	**Global mean**	**Cluster SD**	**Global SD**
**Control- HC**	**n-FW**	2.97	16.50	8.86	7.07	7.49
	**n-RWC**	2.91	96.54	87.55	5.38	9.03
**Control-based HC**	**n-FW**	6.82	144.25	−27.01	85.55	86.69
	**n-GS**	4.27	−3.54	−34.10	16.54	24.35
	**n-RWC**	3.65	0.39	−10.28	8.05	9.97

Table S10. v-test results for Cluster III categorical variables; only significant results are shown. Within-cluster (Mod/Cla), and across-cluster (Cla/Mod) distributions, and global cluster mean of categorical variables are also reported. Variables are ordered by value of v-test.

	**Variable**	**Cla/Mod**	**Mod/Cla**	**Global mean**	**v.test**
**Control- HC**	**HS *A. vulgaris***	100.00	50.00	16.67	2.25
**Control-based HC**	**HS *A. halimus***	72.73	80.00	16.92	4.83
